# Socio-economic predictors of Inuit hunting choices and their implications for climate change adaptation

**DOI:** 10.1098/rstb.2022.0395

**Published:** 2023-11-06

**Authors:** Friederike Hillemann, Bret A. Beheim, Elspeth Ready

**Affiliations:** Department of Human Behavior, Ecology and Culture, Max Planck Institute for Evolutionary Anthropology, Deutscher Platz 6, 04103 Leipzig, Germany

**Keywords:** Arctic Canada, food security, hunting, Inuit, risk-sensitive foraging, socio-ecological systems

## Abstract

In the Arctic, seasonal variation in the accessibility of the land, sea ice and open waters influences which resources can be harvested safely and efficiently. Climate stressors are also increasingly affecting access to subsistence resources. Within Inuit communities, people differ in their involvement with subsistence activities, but little is known about how engagement in the cash economy (time and money available) and other socio-economic factors shape the food production choices of Inuit harvesters, and their ability to adapt to rapid ecological change. We analyse 281 foraging trips involving 23 Inuit harvesters from Kangiqsujuaq, Nunavik, Canada using a Bayesian approach modelling both patch choice and within-patch success. Gender and income predict Inuit harvest strategies: while men, especially men from low-income households, often visit patches with a relatively low success probability, women and high-income hunters generally have a higher propensity to choose low-risk patches. Inland hunting, marine hunting and fishing differ in the required equipment and effort, and hunters may have to shift their subsistence activities if certain patches become less profitable or less safe owing to high costs of transportation or climate change (e.g. navigate larger areas inland instead of targeting seals on the sea ice). Our finding that household income predicts patch choice suggests that the capacity to maintain access to country foods depends on engagement with the cash economy.

This article is part of the theme issue ‘Climate change adaptation needs a science of culture’.

## 1. Introduction

Local foods, whether hunted, fished, gathered, or grown, are critical to the food security and health of Indigenous peoples around the world. Subsistence activities persist in local and Indigenous communities today alongside increased market integration and other economic and social changes of the past century (e.g. [1–3]). In the North American Arctic, mixed cash-subsistence economies have been well-established for decades [4–6] and Inuit continue to harvest and share local foods (called ‘country food’) for their high cultural and nutritional value compared to imported foodstuffs [7–13]. However, access to subsistence resources in Inuit communities in Canada today is strongly affected by the increasing costs of hunting activities. Harvesters are faced with the need to direct money obtained in the cash economy to buy and maintain hunting equipment including motorized boats, all-terrain vehicles (ATV) or snowmobiles, and to purchase gasoline and other supplies [14,15]. The need to engage in wage labour—or, for young harvesters, formal schooling—further reduces the time available for activities on the land [13,16,17]. These recent changes have been linked to different strategies of engagement in the subsistence economy, specifically sharing of country foods (e.g. [13,18,19]), although little research has focused on whether these factors impact land use patterns or the choice of foods to harvest (but see [20,21]).

In addition to socio-economic changes, people in the Arctic are experiencing ecological changes that threaten food security and that require them to adapt their subsistence practises. Climate change affects travel and foraging conditions in numerous ways [22,23]. For example, uncertainty in weather, such as unpredictability of the direction or strength of winds, are major concerns for hunters [24]. Changes in sea ice cover affect access to hunting areas, as well as the behaviour, distribution and migration timing of marine mammals [25,26]. Climate change also impacts access to country food through changes in the availability of key species owing to changes in population health, size or migration routes (e.g. [27–29]), or through wildlife management policies, such as hunting bans or quotas [30,31]. Taken together, novel climate conditions increase the uncertainty about available foraging sites and about the distribution and abundance of resources. These changes not only increase uncertainty but may also affect the probability distribution of returns (risk) of different harvesting activities.

While the archaeological record attests to the ability of Inuit to adapt to climate change, current socio-economic conditions in Inuit communities impose different constraints on—and possibilities for—adaptation than in the past [32]. Worldwide, various cultural adaptations have been described in response to changing climatic conditions ([33–35]). For example, to reduce the risk of harvest shortfalls, the Mikea of Madagascar practise a mix of foraging and farming activities that covary positively and negatively with rainfall [36,37]. Similarly, foragers may pursue a variety of uncorrelated harvest activities (‘Markowitz’s portfolio rule’, [38]). Alaskan Yup’ik and Athabascan people minimize harvest risk by allocating their efforts among a wide variety of species and techniques that differ in their required inputs, by combining rod fishing or pursuits of seals with netting and trapping [39]. However, individual-level risk mitigation strategies that require a variety of equipment, technologies and extensive expertise are not always equally accessible to all. Besides experience and physical capacities, access to cash to finance hunting equipment and supplies may play a role in foraging decisions, and may ultimately constrain peoples’ capacity to mediate climate stressors and maintain involvement in subsistence activities (Buffa *et al*. [40]). Collective behaviour, such as how many other hunters are targeting a resource and the correlation in their activities, may also impact individual risk preferences [41].

The need to consider risk and uncertainty when modelling decision-making, during foraging and in other contexts, has long been acknowledged in biology, anthropology, and economics [42–48]. Theoretical models make predictions about optimal choices of food production to minimize risk or maximize foraging returns under certain conditions, and past research demonstrates that human foragers are sensitive to the mean and variance of success rates of different resources and consider success probabilities in prey and patch choice decisions, often with gender and socio-political objectives affecting risk-sensitivity [2,49–51]. Economic risk preferences have often been argued to be ‘S-shaped’, with both low economic status and high economic status decision-makers willing to accept higher risks because of the potential high gains in case of a success, in comparison to those with intermediate status [42]. In the Arctic today, foraging risks may be reduced through pooling and food sharing, or store-bought foods may buffer variance in foraging returns for Inuit. However, given the reliance of contemporary harvesting on input from the cash economy, it is also relevant to understand how harvesters’ engagement in the cash economy impacts their food production choices and how that affects their ability to engage in different risk reduction strategies and their capacity to adapt to ongoing ecological and economic change.

In this paper, we analyse socio-economic correlates of Inuit harvest production choices, specifically harvesters’ decisions about target prey and foraging method (patch choice). Hunting and sharing of food are complex cultural, social, political, and economic phenomena in mixed cash and subsistence economies, where people engage in wage labour, but traditional harvest activities and food sharing are of great importance for their nutritional health and culture [18,52]. For example, food sharing reinforces social relationships and can affect sharers’ reputations [19]. Patch choice influences food-sharing potential and sharing patterns via the types and quantities of food acquired. On the other hand, the expected outcomes of a successful harvest, with regard to social incentives and political benefits, are themselves likely to affect peoples’ food production choices. Harvest decisions therefore need to be considered in a larger social and economic context. However, relatively little research has directly examined the relationship between socio-economic factors, climate change and foraging decisions. Recent exceptions include Green *et al.* [20], who found that chronic climate stressors most impacted harvest access in two Alaskan Inuit communities, but monetary capital, technology, knowledge, and social relations facilitate continued access to coastal subsistence resources, and Naylor *et al.* [21], who found that the size of the hunting party and time investment are significant to trip productivity, while gasoline expenditures are a poor predictor of Inuit hunting participation and success.

We develop a causal model informed by risk-sensitive foraging theory and past research on Inuit harvesting participation and food security, and model foraging decisions among Inuit hunters by considering both the probability of harvest success within a given patch type and personal, social, and environmental factors that may affect which foraging patches hunters choose. A detailed description of the causes of and constraints on harvesting decisions, including understanding the relationships between socio-economic factors and foraging activities, is key to understanding the persistence of traditional subsistence systems and allows us to make predictions about how people may be differently affected by changing ecological conditions.

## 2. Methods

### (a) Description of the study site

Kangiqsujuaq is an Inuit settlement on the coast of the Hudson Strait in Nunavik. At the time of data collection in 2013–2014, there were 146 Inuit households and approximately 700 residents. Like other contemporary Arctic villages in North America, Kangiqsujuaq has a mixed cash and subsistence economy, where traditional subsistence activities (hunting, gathering and fishing) and food sharing practises persist even though a large proportion of community members work for wages [13,17]. Food insecurity is high in Canadian Arctic communities, and past research has shown that secure access to store foods in Kangiqsujuaq is predicted by household income [53]. Harvest production is, to some extent, correlated with income; but harvest production is also impacted by the type of employment held by harvesters, family composition and access to resources through social networks, among other factors [19,54,55].

Country foods, whether fished, hunted or gathered, represent roughly 10% of calories consumed by Kangiqsujuarmiut, and are both culturally meaningful [56] and disproportionately important for nutrition [57,58]. Nearly all households in Kangiqsujuaq participate in subsistence activities at least occasionally. Nevertheless, a large proportion of the village’s total country food is harvested and distributed by a relatively small proportion of households in the community [19]. Some important country foods include caribou, snow and Canada geese, ptarmigan, beluga whale, ringed seal, Arctic char, and mussels. Beluga in particular is a preferred and culturally significant food [30]. A variety of other foods are occasionally taken (e.g. lake trout, eider ducks, clams, sculpin, eggs and berries), while a number of possible foods are typically not consumed or taken by hunters, owing to high handling costs or taste preferences [14]. For instance, fox are trapped for fur but are not eaten, and harp seal are usually fed to dogs. In this paper, we only focused on harvest activities and returns that are consumed by Inuit, ignoring yields in other currencies (e.g. the fur of some animals including fox and seal are sometimes sold).

### (b) Data collection

The data analysed here were collected as part of a mixed-methods research project focused on Inuit subsistence and food security in Kangiqsujuaq (Nunavik, Quebec) [59]. Between October 2013 and July 2014, E.R. recorded details of all harvesting activities by members of eight Inuit households from different socio-economic backgrounds, during fortnightly interviews. Details recorded in these interviews include the Inuktitut name of the destination (toponym), the harvester’s target species or goal for the trip, number and identity of companions on the trip, the mode of transport and an estimate of gasoline used, and what and how much was harvested (if anything). Some additional harvesting trips were recorded during participation observation (foraging follows) and in occasional interviews between July 2013 and July 2014. Household surveys conducted with 110 households in the village provided information on household composition, economic activities, food security and food sharing activities. Interviews were conducted in English or Inuktitut, by E.R. and a local research assistant.

### (c) Classification of foraging patches

In Kangiqsujuaq, country foods are produced through hunting, fishing and gathering (e.g. for mussels or berries). Most activities require motorized transportation to access harvesting areas, although some activities can be undertaken near the community, notably gathering and some fishing. Boats, ATV or snowmobiles are used to travel depending on the destination and season. The availability of different country foods varies seasonally. Following Smith’s [14] analysis of foraging strategies in Inukjuaq (Nunavik, on the Hudson Bay Coast), we classified foraging patches into general categories that reflect habitat (marine or terrestrial), seasonal differences in availability of harvest areas (on ice or ice-free), target species and/or harvest techniques.

Table 1 provides an overview of the foraging patches; below we provide a general description of each patch. Detailed descriptions of foraging activities that are similar to those undertaken in Kangiqsujuaq can be found in Smith [14]. The primary subsistence activities in the winter months (starting when the sea ice is thick enough to travel on) include hunting on the ice floe edge and inland net ice fishing. Relatively few hunters today participate in harvesting during the coldest and darkest winter months (December to February). When the days get longer and warmer in the spring months (usually starting in April), nearly all community members, including women and children, participate in spring jig-fishing at ice-covered lakes. Other spring activities include hunting for ptarmigan and geese. Both spring and winter harvesting usually involve travel by snowmobile. Summer harvesting begins after ice break-up in late June/early July and is usually undertaken by boat or ATV. During the ice-free months, the primary terrestrial activities include hunting for caribou and birds, and, in the late summer, foraging for berries. Ice-free season marine hunting includes hunting seals and migrating beluga in bays and coastal areas. We group summer and autumn months together, as the activities pursued are broadly similar. The main difference in these seasons is that Arctic char migrate from the sea to lakes in the early autumn and then return to open waters after the early summer thaw. As such, fishing in the summer is a marine activity while in the autumn it takes place at inland lakes. Tidal zone activities primarily involve hand-collecting mussels at low tide, either in temporary caves under the tidal ice or along the coastline. Sculpin, clams, and various other seafood (starfish, urchins) are also taken. Finally, we categorized foraging episodes that were not conducted as a dedicated hunting trip, but that were embedded in trips that occurred primarily for other purposes, as ‘incidental’ hunts.

**Table 1.** Foraging patches of contemporary Inuit hunters across the year, classified by season, prey species and hunting method.

foraging patch	major resources	major methods
winter inland	char	ice netting, rifle snowmobile hunting
winter marine	seal, polar bear	rifle hunting at ice floe edge
spring inland	char, goose, ptarmigan	ice jigging, rifle snowmobile hunting
summer/autumn inland	caribou, char	rifle ATV hunting, lake rod and net fishing
summer/autumn marine	beluga, seal, char	canoe hunting, netting, rod fishing
tidal	mussels	gathering at low tide (especially new and full moon)
incidental	char, birds	embedded in other activities on the land

These patches differ both in their required inputs and in their success rates. For example, tidal mussel picking (in the summertime) is a reliable harvest activity that does not require considerable experience, physical conditioning, or access to expensive hunting supplies. In the wintertime, mussel picking is still reliable but less accessible as open water locations are further from town and going underneath the ice to collect mussels requires an ability to determine if the ice conditions are safe. Other activities have lower success rates and require extensive skills, equipment, and physical strength. For example, winter marine hunts for seals on the ice floe edge are dangerous, require considerable knowledge of sea ice and substantial equipment (including a snowmobile towing a sled with a small boat), and have a low success rate: only 20–25% of trips result in a successful harvest.

### (d) Data analysis

We analysed foraging trip data using two Bayesian mixture regressions to estimate determinants of (i) patch choice and (ii) within-patch success. For each hunt *i*, we model the choice of each patch *X*_*i*_ = *k* from *k* ∈ {1, 2, …, 7} with a categorical distribution, in which the probabilities across patches follow a softmax function of a linear combination of predictor variables. The softmax function converts a vector of *N* real numbers into a vector of *N* real values that sum to 1, by applying the standard exponential function to each element and normalizing them into a probability distribution. Similarly, the probability of success (*Y*_*i*_ = 1) for each hunt *i* is modelled by logistic regression, conditional on a linear combination of factors, including the patch chosen in the *i*th hunt, *X*_*i*_.

**Figure 1.**
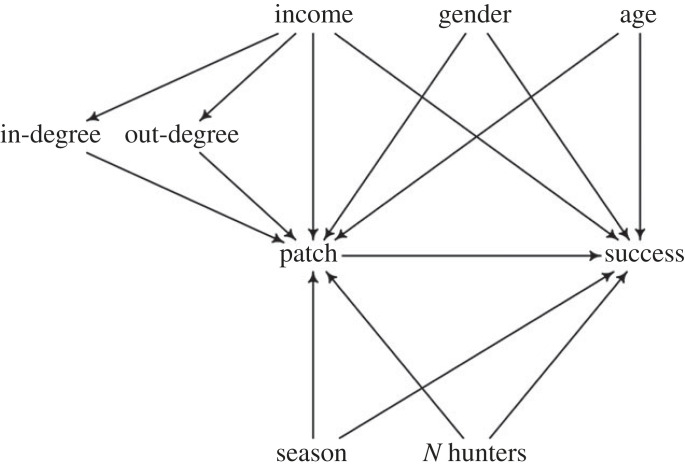
Directed acyclic graph (DAG) used to identify confounders and inform variable selection in the patch choice regression model. Causal assumptions are represented as directed arrows.

We represent our *a priori* causal assumptions using a directed acyclic graph (DAG), which informed variable selection for the statistical analysis (figure 1). For each variable considered, we describe our assumptions and detail how we incorporated them into our statistical model. First, per definition of our patches, season affects which patches can possibly be chosen. In our models, we fitted varying intercepts for each patch type’s probability of choice and success by season (ice season, i.e. winter and spring, and ice-free season) to account for heterogeneity of foraging strategies by time of the year. To capture varying expectations for the probability of patch choice and within-patch success for men and women (owing to differences in experience, for instance), we added harvester’s gender as a varying intercept term for each patch type.

Following latent age-varying skill models of harvest production in hunter–gatherer communities [61,62], we included harvester’s age as a variable to account for varying expectations of physical strength, proficiency and family provisioning responsibilities for different cohorts of harvesters. We fitted a latent variable Gaussian Process model using the following age categories: younger than 30 (less experienced hunters, often with very young children), 30–40 (experienced hunters, often with children in middle-childhood or adolescence), 40–50 (experienced hunters, often with adult children) and older than 50 (experienced hunters, possibly reduced physical fitness).

To estimate potential social effects on harvesters’ patch choice and harvest success, we included information on their position in the community food sharing network, and the number of people who went on the foraging trip as variables in our models. Having many incoming ties in the food sharing network might increase peoples’ willingness to choose patches with a low success probability because a potentially unsuccessful trip could be buffered by receiving shares from other, successful hunters. We might also expect that harvesters who have many outgoing ties (who might also be more skilled hunters) are more willing to hunt in patches with a high risk of failure, if these patches yield large harvests that can be widely shared. Therefore, we included household-level measures of the number of ties in the food sharing social support network in our models (in-degree and out-degree). Here, these are calculated on the basis of sharing reports made by all survey participants excluding the hunter’s own household. This allows us to calculate the in- and out-degree of hunters from all households, even if they did not participate in the survey. These data were collected near the beginning of the data collection period and questions targeted longer-term sharing relationships, and as such reflect existing sharing network position and rather than network ties that resulted from the foraging trips we observed. Nevertheless, care is required in interpreting the effect of the network measures as they also correlate with income and potentially with aspects of skill or experience that are not captured by age or gender variables.

The number of companions on a harvesting trip may affect patch choice and the probability of a successful hunt through a number of mechanisms. For instance, travelling in groups increases safety (although it may slow down some travellers), can facilitate finding and capturing prey, and people may share vehicles or sleds for transportation [21,63]. However, the optimal group size for Inuit hunters varies by hunt type (see discussions of optimal foraging theory in [63]).

Finally, we included household income as our main variable of interest in both the patch choice model and the harvest success model. Income is expected to play an important role in determining the choice of harvest activities and active participation in food sharing, because it relates to the money available to invest into hunting and necessary supplies. To address missing income data for one of the households, we used Bayesian imputation and averaging methods, in which each missing data point for the 35 hunts by this household’s three hunters was given a Normal prior probability with a Normal(0.5, 1) mean and Cauchy(0, 1) standard deviation.

Our statistical modelling was conducted in Stan, v. 2.21.0 (Stan Development Team [64]). For details on the model specifications, Markov chain Monte Carlo (MCMC) settings, and a reproducible example with simulated, anonymous data, see the supplementary code files provided in a Github repository associated with the manuscript. Visual inspection of MCMC diagnostic plots (traceplots), effective sample size and R-hat convergence diagnostic transitions suggest that the chains have mixed well.

## 3. Results

The data consist of 281 foraging episodes involving 23 Inuit harvesters from 13 households. Our sample includes 47 trips to patches classified as winter inland, 38 winter marine, 72 spring inland, 21 summer/autumn inland, 75 summer/autumn marine, 20 tidal and eight incidental episodes. The number of trips analysed per harvester ranged from 1 to 71 (median: 7.5, 25–75 per cent quantiles: 2–17.75). Harvesting groups usually consist of 2–4 people (25–75% quantiles; median: 3). The majority of foraging trips included in our analysis were conducted by men (228 out of 281 trips). Groups of harvesters may have been mixed-gender and from multiple households, but we analyse patch choice and harvest returns from the perspective of the focal harvester (usually the person who reported the activity).

The regression models estimate the posterior probabilities of a patch being chosen and the probability of a successful harvest for each of the patch types. We primarily focus on the role of income in shaping patch choice. Additional plots focusing on other variables in the DAG are presented in the electronic supplementary material. We also provide details of the model coefficients in the electronic supplementary material. However, because of correlations among the variables, especially income and number of sharing network ties, we focus only on model predictions and do not interpret the model coefficients directly. Instead, we present model predictions for hypothetical individuals with realistic combinations of traits.

**Figure 2.**
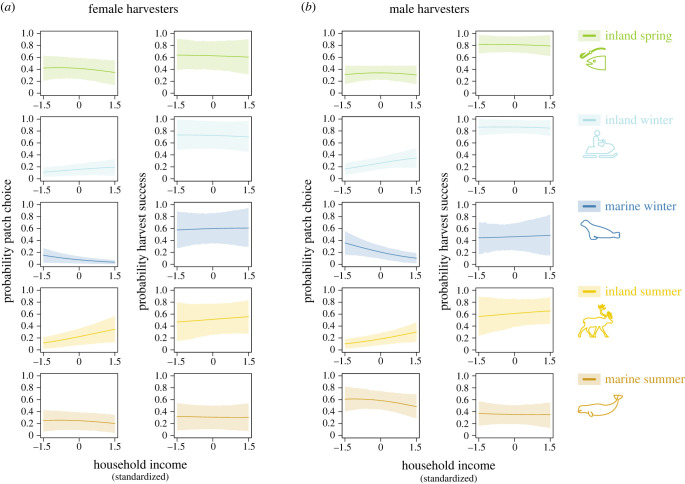
Standardized effects of household income on (*a*) women’s (*b*) men’s patch choice and harvest success (left and right panel, respectively). Posterior distributions (mean and 89% highest posterior density) for probability of patch choice and within-patch success as a function of household income (standardized; the range of values is not extrapolated but matches observed income ranges). All data are shown for a 40–50-year-old person with an average number of giving and receiving ties in the food sharing network (i.e. out-degree and in-degree set to four ties). Only the most common patch types are shown.

We find that household income affects patch choice among Inuit harvesters in a number of ways (figure 2). Given the high costs of hunting supplies and fuel for transportation [5,65], we expected that income would be positively associated with choosing patches that are more cost-intensive and with the probability of a successful harvest in those patches. In particular, marine summer hunting requires access to a motorized boat, ATVs or snowmobiles are required to reach inland patches, and success may be impacted by search distance, which can be limited by fuel costs. By contrast, an activity like mussel-picking (tidal patch) is likely to be equally accessible and productive for harvesters regardless of income. We find that, while an increase in household income is indeed associated with an increased probability of choosing inland patches, both in winter and summer, high-income harvesters are less likely than low-income harvesters to choose winter marine patches (figure 2). Overall, our results indicate that high-income harvesters have a somewhat more balanced portfolio of harvesting activities, although they are less likely to participate in winter marine hunting. Contrary to our expectation, we did not find evidence that income predicts within-patch harvest success, i.e. once a patch was chosen (figure 2).

Among Kangiqsujuarmiut, income is correlated with the number of connections in the food sharing network [19]. Households with a high number of food-receiving ties tend to be low-income households, whereas high-income households usually have a high out-degree (Pearson correlation coefficient for income and in-degree: *r* = −0.22, for income and out-degree: *r* = 0.39; based on 12 households included in our analyses, with known income and number of network ties). The correlations of variables reported here differ slightly from those reported for all 110 surveyed households (*r* = −0.04 and *r* = 0.48, respectively; [54, table 4.2]), though household degree was calculated using different methods in these analyses (here, we exclude self-reported edges). The number of incoming and outgoing ties in the food sharing network are not strongly correlated in our smaller sample of harvesters (*r* = −0.07, *n* = 13 households), reflecting purposive sampling of households in this subset to represent a broad variety of harvesters. Generally, with the notable exception of some elders, a higher in-degree is associated with a higher out-degree, while people with a high out-degree may have a wider range of incoming ties. We find that a harvester’s number of food-sharing connections is linked to their patch choice and harvest success probabilities, especially for marine foraging, which includes seal and beluga hunting (electronic supplementary material, figure S1). Harvesters with a low number of food-receiving ties are more likely to choose marine patches compared to those with a higher in-degree. Interestingly, although people with a high in-degree are less likely to choose winter marine trips, their within-patch success probability is high (electronic supplementary material, figure S3).

Women are more likely than men to chose tidal and inland patches instead of marine patches; these patches also tend to have a higher chance of success (figure 2). We do not find evidence that a harvester’s age plays an important role in determining their patch choice (electronic supplementary material, figure S5). Our dataset contains fewer harvest trips by older people than younger people, which means that the estimates of effect sizes suggest that younger harvesters are slightly more likely to choose any given patch type compared to older people. However, across patch types, none of the age classes have very different preferences for patch types (electronic supplementary material, figure S2). The number of people involved in a trip is positively associated with spring inland harvest activities, such as jig fishing at lakes and hunting for birds, which are conducted by young and old community members regardless of gender (electronic supplementary material, figures S1 and S2).

**Figure 3.**
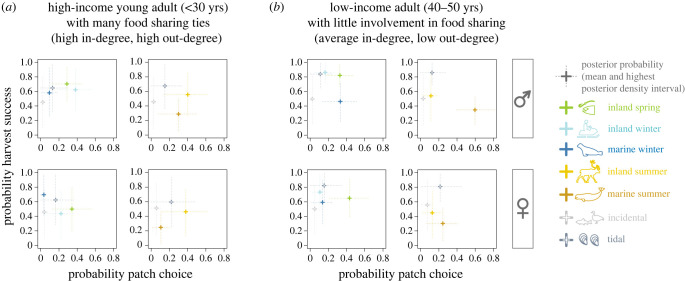
Posterior predictive probabilities of patch choice and within-patch success for harvesters with different socio-economic profiles: (*a*) young adult with a higher-than-average household income and good embedding in the food sharing network (in-degree: 8, out-degree: 8), and (*b*) 40–50-year-old person with a low income and who receives more food than they give to others (in-degree: 4, out-degree: 1). The top row shows data for men, the bottom row shows data for women. Data of patches that could be chosen during the snow and ice period are shown in green and blue tones (left panel, respectively), patches that could be chosen in the ice-free season are shown in yellow tones (right panel, respectively), and data for incidental and tidal foraging trips, which could be made throughout the year, are plotted in grey tones. Shown are the mean and the credible intervals (highest posterior density interval with 89% probability mass) of samples from the posterior distributions. The mean of the prior predictive distribution for patch choice is 0.14 (0.89% interval: 0.05–0.24), and the mean of the prior predictive distribution for harvest success is 0.50 (0.89% interval: 0.31–0.69).

To better understand harvest production choices for people with different profiles (age, gender, income and number of ties in the food sharing network), we examine the relationships between the probability of a patch being chosen and within-patch success for a set of realistic but hypothetical harvesters (figure 3). For a more systematic representation of predictions for hypothetical harvesters see the electronic supplementary material, figure S3. A positive correlation between choice and success probabilities would indicate that harvesters reduce the risk of a failed trip, by choosing patches with the highest probability of being successful most often. However, our results indicate that the probability of success alone does not explain Inuit foraging patch choices. For example, ice-free marine hunts, which include beluga whale hunting, stand out for having a surprisingly high probability of being chosen given the moderate probability of a harvest success for this patch, especially in adult (40–50-year-old) men with low in-degrees (electronic supplementary material, figure S3), whereas tidal mussel picking has a low probability of being chosen across harvester profiles, despite the very high probability of harvest success (figure 3; electronic supplementary material, figure S3).

## 4. Discussion

There is a growing literature on the relationship between wage labour and harvest production in Inuit communities [7,13,17,39,53,66–69]; our analysis further describes how these factors shape harvesters’ use of the land and its resources. Our results demonstrate that Inuit patch choice is affected by socio-economic status, notably gender, income, and embeddedness in the sharing network (which, for out-degree, also proxies past productivity and skill). For example, men choose marine hunts in the ice-free season surprisingly often, considering the moderate success rate of this activity. This patch category includes hunts for migratory beluga whales, a highly valued large prey that is often widely shared. Social incentives might influence decisions about where to go hunting or with whom. A successful beluga hunt, for example, is culturally important and provides social benefits through food sharing, which can improve a hunter’s social or political standing [30,70–75]. Similarly, previous work has described an assortment of hunters by harvest productivity through preferential sharing of country foods among households with similar socio-economic status [19].

Consistent with past ethnographic descriptions, women’s harvest activities are particularly focused on spring fishing, tidal gathering and summertime harvesting activities. Tidal gathering and spring fishing are also among the most predictable patches. Women were never observed in the winter marine patch. We note, however, that our sample of women’s foraging trips is relatively small. Women’s choices are additionally determined by cultural and situational factors (including division of labour, family composition, and birth order) which shape opportunities for women to learn to hunt. As such, patterns of patch choice among women reflect these factors rather than a greater risk aversion compared to men *per se*. In fact, some women in Kangiqsujuaq are considered to be accomplished hunters, the most well-known among them being the author and sculptor Mitiarjuk Nappaaluk. Indeed it was on the basis of work in Kangiqsujuaq that Saladin d’Anglure [76] proposed the ‘third gender’ of the Inuit, although recent work by Kallaleq scholar Jessen Williamson suggests that Inuit conceive of the soul as genderless [77].

While household income has little to no effect on either women’s or men’s within-patch success probability across patches, our results show that income affects Inuit patch choice, suggesting that harvesters differ in their interests and motivation when deciding where and what to hunt, or that they have different means to accessing patches. Increased income is linked to an increased probability of choosing inland patches, but more affluent hunters are less likely to participate in marine activities during the snow and ice season. Marine hunts across the ice floe edge are considered a particularly dangerous activity, whereas tidal and inland patches require less specialized knowledge than marine patches (i.e. the ability to drive a boat in rough or shallow waters, or sea ice knowledge).

It is important to note that all data included in our analyses were from trips that were actually made and therefore there is a selection bias in the observed harvest success rates (compared to the hypothetical case of harvesters being assigned randomly to patches). As such, the patch choice preferences of some harvesters are not included in our analyses, because they were less likely to be observed hunting (perhaps owing to socio-economic constraints). Additionally, the patch choices we observed may not reflect risk-preferences *per se*, as opposed to simply reflecting constraints on choices (e.g. lack of a boat). Consequently, we cannot infer from our data whether the observed patch preferences reflect risk-seeking as opposed to risk-avoidance behaviours.

The impacts of climate change are manifold, and people are not expected to be equally affected by, or able to moderate, its consequences. It is becoming increasingly clear that impacts of the cash economy, including economic barriers to harvesting, may intensify socio-economic inequalities and affect the long-term resilience of Arctic food systems [32,78,79]. Sea ice loss, erosion and increasing variability in weather conditions are some of the consequences of climate change that affect the accessibility of the land, the sea ice and open waters [20]. While altering land use patterns may facilitate involvement in subsistence activities ([21,80]), some people cannot afford the high costs of fuel or buying and maintaining vehicles for safe transportation to alternative harvest areas. Similarly, people may not be able to target alternative prey owing to a lack of access to hunting supplies. Spreading harvest efforts across different activities may reduce the risk of shortfalls if some patches become less productive or available, but such diversification requires time, skill and knowledge, in addition to the ability and willingness to finance traditional subsistence activities (e.g. [20,21]).

Understanding the relationships between socio-economic factors and foraging activities allows us to develop informed predictions about how people will be affected by changing conditions. For example, retreating sea ice and longer ice-free periods owing to a warming climate will make hunters and fishers more reliant on boats to reach harvest areas. Already during data collection in 2013, some hunters reported that the availability of the winter marine patch was delayed by poor ice conditions, making seal hunting dangerous. The available alternative patch during this season is primarily inland fishing, leading to potential reductions in the diversity of species caught. Revisiting our hypothetical individuals from figure 3 with this scenario in mind indicates that younger, low-income men would be the most affected by the reduced availability of winter marine patches. On the other hand, the hunting activities of high-income young adults with many in- and out-going ties in the food sharing network, and women who rarely use marine patches but prefer inland harvest activities in the winter and spring months, are unlikely to be strongly directly affected by sea ice becoming inaccessible for winter hunting, though one might expect indirect effects, for instance owing to the need to compensate for lowered food availability. We find that high-income harvesters generally have a broader portfolio of harvesting activities than low-income harvesters, which suggests that they may have a greater capacity to adapt to changes in the accessibility of harvest resources.

While most research on climate change adaptation in the Arctic has focused on identifying behaviours or practises that harvesters could adopt in order to mitigate the effects of climate change [79], social and economic structures also constrain what options are feasible for actors [81,82]. Inuit in Kangiqsujuaq themselves identify costs of living and of hunting equipment and supplies as major barriers to harvesting today [79], and our results echo their perception: socio-economic factors impact harvesting strategies and thus may constrain adaptation to climate change for some community members more than others. In Kangiqsujuaq, community leaders are concerned to support food security and access to country food for all residents. Detailed analyses of the impacts of social, economic and ecological factors on behaviour can provide communities with the knowledge needed to effectively work towards these goals.

## Data Availability

Simulated data and code to replicate the analyses presented in the main text are available from the Github repository: https://github.com/fhillemann/MSrepo_harvest_patch_choice.git [83], and from the Dryad Digital Repository: https://doi.org/10.5061/dryad.k3j9kd5dv [84]. This is part of the Climate Change Adaptation Needs a Science of Culture data portal from the Dryad Digital Repository: https://doi.org/10.5061/dryad.bnzs7h4h4 [85]. The repository includes Stan files to reproduce the patch choice model and the harvest success model and R code to simulate and analyse harvest trip data.
